# Effects of osteopontin inhibition on radiosensitivity of MDA-MB-231 breast cancer cells

**DOI:** 10.1186/1748-717X-5-82

**Published:** 2010-09-17

**Authors:** Antje Hahnel, Henri Wichmann, Matthias Kappler, Matthias Kotzsch, Dirk Vordermark, Helge Taubert, Matthias Bache

**Affiliations:** 1 Department of Radiotherapy, Martin-Luther-University Halle-Wittenberg, Dryanderstr.4, 06110 Halle, Germany; 2 Department of Oral and Maxillofacial Plastic Surgery, Martin-Luther-University Halle-Wittenberg, Ernst-Grube-Str.40, 06120 Halle, Germany; 3 Institute of Pathology, Dresden University of Technology, Fetscherstr.74, 01307 Dresden, Germany

## Abstract

**Background:**

Osteopontin (OPN) is a secreted glycophosphoprotein that is overexpressed in various tumors, and high levels of OPN have been associated with poor prognosis of cancer patients. In patients with head and neck cancer, high OPN plasma levels have been associated with poor prognosis following radiotherapy. Since little is known about the relationship between OPN expression and radiosensitivity, we investigated the cellular and radiation induced effects of OPN siRNA in human MDA-MB-231 breast cancer cells.

**Methods:**

MDA-MB-231 cells were transfected with OPN-specific siRNAs and irradiated after 24 h. To verify the OPN knockdown, we measured the OPN mRNA and protein levels using qRT-PCR and Western blot analysis. Furthermore, the functional effects of OPN siRNAs were studied by assays to assess clonogenic survival, migration and induction of apoptosis.

**Results:**

Treatment of MDA-MB-231 cells with OPN siRNAs resulted in an 80% decrease in the OPN mRNA level and in a decrease in extracellular OPN protein level. Transfection reduced clonogenic survival to 42% (p = 0.008), decreased the migration rate to 60% (p = 0.15) and increased apoptosis from 0.3% to 1.7% (p = 0.04). Combination of OPN siRNA and irradiation at 2 Gy resulted in a further reduction of clonogenic survival to 27% (p < 0.001), decreased the migration rate to 40% (p = 0.03) and increased apoptosis to 4% (p < 0.005). Furthermore, OPN knockdown caused a weak radiosensitization with an enhancement factor of 1.5 at 6 Gy (p = 0.09) and a dose modifying factor (DMF_10_) of 1.1.

**Conclusion:**

Our results suggest that an OPN knockdown improves radiobiological effects in MDA-MB-231 cells. Therefore, OPN seems to be an attractive target to improve the effectiveness of radiotherapy.

## Background

OPN is a secreted phosphoglycoprotein (SSP1) expressed by osteoclasts and osteoblasts, epithelial cells, activated immune cells and tumor cells. OPN is a member of the SIBLING (Small integrin-binding ligand N-linked glycoproteins) protein family and contains a characteristic RGD-motif that mediates the binding to α_ν_β-integrin receptors and a thrombin cleavage side, which releases a CD44-binding domain. Several signaling cascades such as the NF-kB/IkBα/IKK pathway, PI3'-kinase/Akt pathway and the MAPK-dependent pathway are activated by the interaction between OPN and membrane receptors and take part in a variety of normal and pathologic processes. Therefore, the OPN protein influences processes that are important for tumor progression and metastasis (e.g., proliferation, cell motility, migration, invasion and apoptosis; reviewed in [1,2]).

In various studies, OPN overexpression has been linked to high invasive and metastatic potential, recurrent disease and poor prognosis for cancer patients [3-6]. Moreover, a recent immunohistochemical study of prostate cancer tissues demonstrated that OPN protein expression is not increased after radiotherapy. However, patients with aggressive prostate cancer had significantly higher OPN protein expression, which was associated with decreased freedom from biochemical failure [7]. Furthermore, a study of rectal cancer showed that patients who received successful therapy had much lower pre-therapy OPN levels compared to patients who later developed metastases [8]. OPN has been discussed not only as tumor marker but also as a marker of hypoxia [9,10]. In a previous report from our group, immunohistochemical OPN expression was found to be associated with low tumor oxygenation in advanced head and neck cancer treated with radiotherapy or chemoradiation [11]. Similarly, Le and co-workers reported that high OPN plasma levels are associated with tumor hypoxia in head and neck squamous cell carcinomas and correlate with poor clinical outcome [12]. In addition, a clinical study by Overgaard and co-workers [13] found that high OPN plasma concentrations are associated with a poor prognosis after radiotherapy for patients with head and neck cancer. However, prognosis of patients with high OPN plasma levels could be improved after treatment with the hypoxic radiosensitizer nimorazole [13]. It is known that tumor hypoxia is a major determinant of radioresistance. However, little is known regarding the relationship between OPN expression levels in tumor cells and their radiosensitivity. Therefore, it is important to investigate OPN and its role in cancer progression to improve the opportunities of cancer therapy, especially the effectiveness of radiotherapy.

It is well known that OPN plays an important role in breast cancer. Several studies prove that OPN is overexpressed in breast cancer and that this correlates with high malignancy, poor prognosis and survival [3-5,14,15]. Accordingly, we chose the MDA-MB-231 cell line to investigate the effect of an OPN knockdown and irradiation on migration, apoptosis and clonogenic survival. Primary tests showed that the MDA-MB-231 cell line is a radiation insensitive cell line (dose response curve is not shown). We determined an SF_2_-value of 0.60. Other groups described similar SF_2_-values with an average of 0.65 (SF_2 _= 0.82 [16]; SF_2 _= 0.63 [17]; SF_2 _= 0.5 [18]).

To determine the influence of OPN on migration, apoptosis, clonogenic survival and radiosensitivity, we reduced the OPN mRNA level in MDA-MB-231 breast cancer cells by transfection with OPN specific siRNA.

## Methods

### Cell culture conditions

The human breast cancer cell line MDA-MB-231 was grown as a monolayer in RPMI 1640 containing 25 mM HEPES and L-glutamine (Lonza, Walkersville, USA). The medium was supplemented with 10% fetal calf serum (FCS) (PAA, Cölbe, Germany), 1% pyruvate (Invitrogen, Karlsruhe, Germany), 185 units/ml penicillin (Invitrogen), and 185 μg/ml streptomycin (Invitrogen), and cells were cultured in a humidified atmosphere of 3% CO_2 _at 37°C. All experiments were performed with cells in logarithmic growth phase.

### Treatment with OPN siRNAs and irradiation

Two double-stranded OPN siRNA oligonucleotides (Mix, OpnS) and a nonsense siRNA (negative control) were transfected using INTERFERin™ reagent as recommended by the manufacturer (Polyplus Transfection Illkirch, France). The cells (4-5*10^5 ^cells) were plated overnight at 37°C, 3% CO_2 _and then transfected with 100 nM of either nonsense non-targeting siRNA or target-specific siRNAs to knockdown OPN for 24 h and 72 h. The siRNA oligonucleotide sequences are shown in Table 1.

**Table 1:** siRNAs

target-mRNA	siRNA	sequence 5'→3'	localization	source
nonsense	Lu GL2	5'-CGTACGCGGAATACTTCGA-3'		
osteopontin	Mix (SMART pool)	5'-CAUCUUCUGAGGUCAAUUA-3' 5'-UGAACGCGCCUUCUGAUUG-3' 5'-CCGAUGUGAUUGAUAGUCA-3' 5'-GGACUGAGGUCAAAAUCUA-3'	1091-2009 797-814 938-956 661-679	Dharmacon Inc. (Chicago, IL, USA)
osteopontin	OpnS	5'-GAACGACUCUGAUGAUGUA-3'	480-498	[32]

Sequences and localization of siRNAs used in this study that correspond to mRNA sequences of OPN [GenBank: NM_001040058]

Furthermore, the cells were irradiated in tissue culture flasks (Greiner, Frickenhausen, Germany) at 2, 4 or 6 Gy 24 h after OPN siRNA transfection. Irradiation at 0 to 6 Gy was accomplished in logarithmically growing cultures with 6 MV photons and adequate bolus material on a SIEMENS ONCOR (Erlangen, Germany) linear accelerator at a dose rate of 2 Gy/min. Referring to the fractionated daily dose in therapy treatment and DMF_10_-value of the MDA-MB-231 cell line, we have chosen a radiation dose of 2 Gy and 6 Gy, respectively. At 1 h and 48 h after irradiation, cells were processed for RNA and protein extraction, clonogenic assays (1 h) and migration and apoptosis assays (48 h).

### Quantitative real-time RT-PCR (qRT-PCR)

Total RNA was isolated using the RNeasy^® ^Mini Kit as recommended by the manufacturer (Qiagen, Hilden, Germany). For hybridization, 1 μg of RNA was incubated with random primers (150 ng/μL) at 70°C for 10 min followed by addition of 5× first strand buffer, 0.1 M DTT, 2.5 mM dNTPs and SuperScript™ II reverse transcriptase (200 U/μl) (Invitrogen). The reaction conditions were: 20°C for 10 min, 42°C for 80 min and 95°C for 10 min.

All qRT-PCR reactions were performed on a Rotorgene RG-6000 (LTF, Wasserburg, Germany) using the QuantiTect SYBRGreen PCR Kit (Qiagen). For each PCR reaction, 1 μl of cDNA was added to SYBRGreen Quantitect 2×, PCR primers (20 μM) and aqua bidest in a total volume of 15 μl. As a negative control, we used a no-template reaction. The primers used are cited in Table 2. HPRT (hypoxanthineguanine phosphoribosyltransferase) served as a housekeeping gene and for control of cDNA integrity. PCR conditions were: 95°C for 15 min followed by 40 cycles of denaturation for 30 s at 95°C, hybridization for 30 s at 60°C, extension for 30 s at 72°C, a final step for 30 s at 60°C and a melting curve program (65-95°C with a heating rate of 0.2°C/s). RNA was isolated as well as cDNA was generated and quantified from three independent experiments.

**Table 2:** Primers for quantitative real-time RT-PCR

gene	primer	sequence 5'→3'		localization
HPRT	HPRT fw	5'-TTGCTGACCTGCTGGATTAC-3'	sense	309-328
	HPRT rev	5'-CTTGCGACCTTGACCATCTT-3'	antisense	551-570
OPN	OPN fw	5'-TGGCCGAGGTGATAGTGTG-3'	sense	555-573
	OPN rev	5'-CGGGGATGGCCTTGTATG-3'	antisense	686-703

Primer sequences and the localization of the primer binding side in the corresponding mRNA transcript

### Western blot hybridization

The cells were lysed in RIPA buffer (50 mM Tris-HCl pH 7.4, 200 mM NaCl, 1 mM EDTA, 1 mM EGTA, 1% Triton X-100, 0.25% desoxycholate, 1:100 phosphatase inhibitor, 1:100 proteinase inhibitor) followed by ultrasonic homogenization. The conditioned medium (serum-free RPMI) was harvested after 24 h and 48 h and spun at 1,300 rpm for 10 min to remove cell debris. The supernatant was concentrated using Amicon^® ^Ultra Centrifugal Filters (Millipore, Billerica, MA, USA) with a 3 kDa cut-off.

Equal amounts of protein (15-20 μg/lane) were electrophoresed on 4-12% Bis-Tris gradient gels (Invitrogen) under reducing conditions and transferred to PDVF membrane (Millipore GmbH, Schwalbach, Germany). The membrane was blocked with 10% non-fat milk in TBST (50 mM NaCl, 30 mM Tris-HCl pH 8.0, 0.1% Tween) for 1 h and probed with polyclonal rabbit anti-human OPN (1:2,000, 0-17, IBL, Hamburg, Germany), rabbit anti-human cleaved PARP (poly-(ADP-ribose)-polymerase) (Asp214) (1:2,000, Cell Signaling, Danvers, MA, USA) and mouse anti-β-actin (1:5,000, Sigma, Steinheim, Germany) at 4°C overnight. The membrane was washed three times with TBST buffer for 7 min followed by incubation with HRP-conjugated secondary antibodies (DAKO, Hamburg, Germany) diluted 1:5,000 in TBST containing 10% non-fat milk for 1 h at room temperature. After further washing steps (three times with TBST buffer and one time with TBS), the immunocomplexes were visualized by ECL or ECL Plus Blotting Detection System (Amersham, Freiburg, Germany). We analyzed the conditioned medium of two independent experiments and the protein data of three independent experiments.

### Clonogenic survival assay and radiosensitivity

The cells were trypsinized 1 h after irradiation, and different numbers of cells (100-10,000), depending on treatment and irradiation dose, were seeded into 25-cm^2 ^cell culture flasks. The cells were cultured in RPMI supplemented with 10% FCS in a humidified atmosphere of 3% CO_2 _at 37°C. The cells were incubated for two weeks and then fixed with paraformaldehyde (Sigma), and colony formation (colonies of ≥50 cells) was visualized by staining with 10% Giemsa solution (Sigma). The number of colonies was counted to determine the survival fraction (SF), determined as the ratio of number of colonies formed by irradiated cells to the number of colonies formed by non-irradiated cells. The enhancement factor was determined as the ratio of the survival fraction of OPN siRNA-treated cells to nonsense siRNA-treated control cells. The DMF_10 _is the radiation dose that characterizes an effect at the survival level of 10% of the colonies. The data represent at least three independent experiments.

### Migration assays

Cell migration was assessed using modified Boyden chambers [19]. Cells (2.0*10^4^) were suspended in 300 μl of RPMI without FCS and were added to the upper chamber (membrane filter with 8 μm pore size), and the bottom chamber was filled with 1 ml of RPMI supplemented with 20% FCS as chemoattractant. The assay was incubated at 37°C in a humidified atmosphere containing 3% CO_2 _for at least 16 h. Non-migrating cells on the upper side of the transwell inserts were removed. The migrated cells on the bottom side of the membrane filter were trypsinized and counted with CASY^® ^DT (Schärfe System GmbH, Reutlingen, Germany). The data represent at least three independent experiments.

Furthermore, we used a wound scratch assay to determine the migration of MDA-MB-231 cells after transfection with OPN siRNA. Cells were grown in 6-well culture plates [19] in RPMI culture medium containing 10% FCS and cultured to 100% confluence. A uniform cell-free area was created by scratching a confluent monolayer with a 200 μl pipette tip. To determine the migration of MDA-MB-231 cells, the wound closure was observed at different time points. The wound scratch assay was also performed in three independent experiments.

### Apoptosis

For quantitative determination of the rate of apoptosis, we analyzed suspended cells and the corresponding supernatant. The cells were fixed with 80% ethanol (Merck, Darmstadt, Germany) and centrifuged on microscope slides at 1000 g for 5 min. After staining with DAPI solution (4,6-diamidino-2-phenylindole dihydrochloride) (Serva, Heidelberg, Germany) and washing with PBS, the cells were covered with ProLong^® ^Gold antifade reagent (Invitrogen). The rate of apoptosis was quantified with a fluorescent microscope at 200× magnification (MC 100 Spot, Zeiss universal microscope, Jena, Germany) by counting 500 cells in separate visual fields (described in [20]). The data represent the results of at least three independent experiments.

### Statistical analysis

The experimental results were checked for normal distribution and therefore analyzed by unpaired Student's *t*-test, where p < 0.05 was considered as an indicator of a significant difference between mean values.

## Results

### Effects of OPN siRNA constructs on mRNA and protein levels with or without irradiation

**Figure 1:**
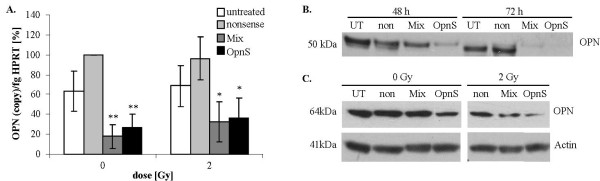
**OPN mRNA and protein levels of either non-irradiated or irradiated MDA-MB-231 cells after siRNA transfection**. **A. Quantitative real-time PCR**: OPN mRNA levels of untreated cells and cells treated with siRNA targeting OPN or nonsense siRNA. Representative values of OPN mRNA levels (72 h after transfection) treated with OPN-specific siRNAs were normalized to those treated with nonsense siRNA. The value of the OPN mRNA level of cells that were treated with nonsense siRNA at 0 Gy was arbitrarily established as 100%. Data represent the average values (± SD) of three independent experiments (* p < 0.05, ** p < 0.001). **B./C. Western blot**: Western blot analyses of OPN with OPN specific antibody 0-17 (IBL). **B**. MDA-MB-231 cells were transfected with siRNA Mix as well as OpnS or with nonsense siRNA (non) for 24 h. Thereafter, MDA-MB-231 cells were incubated with serum-free culture media for another 24 h and 48 h. The Western blot shows the extracellular OPN protein levels (50 kDa) of MDA-MB-231 cells 48 h and 72 h after transfection with OPN specific siRNA Mix and OpnS, with nonsense siRNA (non) and untreated MDA-MB-231 control cells (UT). The Western blot shows one representative result out of two independent experiments. **C**. Intracellular OPN protein levels (64 kDa) of MDA-MB-321 cells 24 h after transfection. Cells were either untreated (UT) or treated with OPN specific siRNA Mix and OpnS or with nonsense siRNA (non) with and without irradiation at 2 Gy. The Western blot shows one representative result out of three independent experiments. Actin served as an internal loading control.

At 24 h and 72 h after transfection, the OPN mRNA level in cells treated with OPN-specific siRNAs (Mix, OpnS) was approximately 20% compared to that in cells treated with control siRNA (nonsense siRNA) (Fig. 1A.). We further studied the OPN mRNA level after treatment with OPN-specific siRNAs and additional irradiation. We found that irradiation alone had no effect on OPN mRNA levels. However, after irradiation at 2 Gy in both Mix and OpnS transfected cells, OPN mRNA levels were found to be reduced to 30% compared to cells treated with control siRNA (Fig. 1A.). These effects could be seen at 24 h as well as 72 h after transfection in combination with irradiation at 2, 4 or 6 Gy (data not shown).

Western blot analysis was used to determine the effects of OPN knockdown on the OPN protein level. Transfection with either Mix or OpnS resulted in a clear decrease in the extracellular OPN protein level (Fig. 1B.). However, a decreased intracellular OPN protein level after siRNA transfection was only partially detectable (Fig. 1C.). Furthermore, our experiments demonstrated that the OPN protein level is reduced in control cells transfected with nonsense siRNA after irradiation at 2 Gy compared to non-irradiated cells. The irradiation-induced inhibition of OPN protein expression was also detected in cells transfected with OPN siRNAs (Fig.1C.).

### Effects of OPN siRNA constructs on migration and induction of apoptosis with or without irradiation

**Figure 2:**
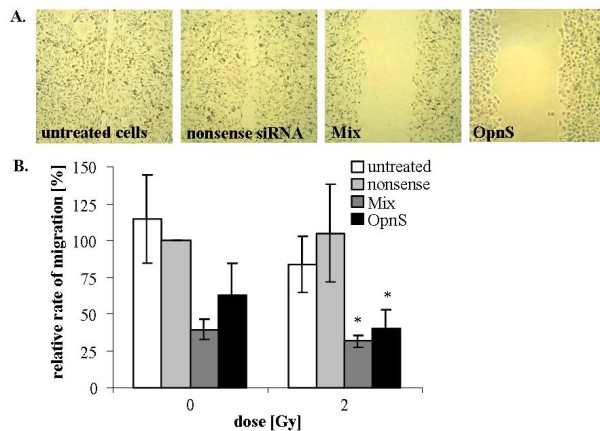
**Migration behavior of either non-irradiated or irradiated (2 Gy) MDA-MB-231 cells after siRNA transfection**. **A. Scratch assay**: Wound scratch assay of MDA-MB-231 cells 24 h after transfection. Untreated cells and cells that were treated with nonsense siRNA were able to close the wound scratch by migration. Cells treated with Mix as well as OpnS did not migrate and were unable to close the wound scratch. **B. Boyden chamber assay**: The migration rate of cells treated with OPN-specific siRNAs was normalized to migration rate of cells treated with nonsense siRNA. Treatment with siRNAs targeting OPN reduced the migration rate in non-irradiated cells as well as in cells irradiated at 2 Gy. The migration rate of cells transfected with nonsense siRNA at 0 Gy was arbitrarily established as 100%. Data represent the average values (± SD) of three independent experiments (* p < 0.05).

**Figure 3:**
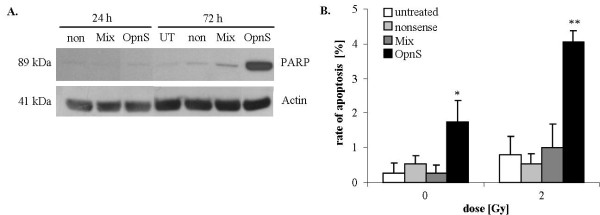
**PARP protein levels and apoptosis rate of either non-irradiated or irradiated cells after siRNA transfection**. **A**. Western blot analysis of PARP with rabbit anti-human cleaved PARP (Asp214) antibody [1] in MDA-MB-321 cells 24 h and 72 h after transfection. The cells were untreated (UT), transfected with 100 nM of either nonsense siRNA (non) or target-specific siRNAs to knockdown OPN (Mix and OpnS). The Western blot shows one representative result out of three independent experiments. Actin served as an internal loading control. **B**. The morphology of DAPI stained cell nuclei was analyzed to quantify the apoptosis rate of MDA-MB-231 cells 72 h after transfection. The diagram shows the apoptosis rate of the cells as a function of treatment and irradiation. A fluorescence microscope was used and 500 cells in several fields of view were counted for each experiment. Data represent the average values (± SD) of three independent experiments (* p < 0.05, ** p < 0.001).

We determined the effects of OPN siRNA and irradiation on the migration rate of MDA-MB-231 cells with the Boyden chamber assay and scratch assay. Cells transfected with siRNA targeting OPN showed reduced migration rates compared to control cells (control and nonsense siRNA). Transfection with Mix resulted in a decreased migration rate to 40% (p = 0.09), whereas the migration rate of cells transfected with OpnS was less than 62% (p = 0.15) compared to the migration rate of cells treated with control siRNA (Fig. 2B.). Similarly, we found a reduced migration rate after transfection with OPN siRNA using the scratch assay (Fig. 2A.). Furthermore, we demonstrated that irradiation at 2 Gy to 6 Gy had no effect on the migration rate (data not shown). However, combination of OPN siRNA transfection and irradiation at 2 Gy resulted in a significant inhibition of migration. After incubation with Mix and 2 Gy irradiation, migration was reduced to 32% (p = 0.03). Additionally, transfection with OpnS and irradiation at 2 Gy attenuated the migration rate to 40% (p = 0.03). Using Western blot analysis, we examined PARP cleavage as an indicator for the induction of apoptosis. However, 24 h after incubation with OPN siRNA, we could not detect any PARP cleavage products using Mix or OpnS. Moreover, Fig. 3A. shows a distinctive accumulation of the PARP cleavage product (89 kDa) 72 h after transfection with siRNA OpnS. However, only OpnS, not Mix, induced apoptosis (Fig. 3A. and 3B.). In addition, we examined the morphology of the cell nuclei to quantify the rate of apoptosis by the use of DAPI staining. The results observed in Western blot analyses were supported by the findings of the quantitative assay. After incubation with OpnS, the apoptosis rate increased from 0.3% to 1.7% (p = 0.04), whereas transfection with Mix had no effect on apoptosis. We found that irradiation alone at 2 Gy did not significantly increase apoptosis in MDA-MB-231 cells (Fig. 3B.). Nevertheless, the combination of OpnS and irradiation at 2 Gy resulted in a significant increase in apoptosis rate to 4% (p = 0.0001). In contrast to that, incubation with Mix and irradiation at 2 Gy had no effect on apoptosis.

### Effects of OPN siRNA on clonogenic survival and radiosensitivity

**Figure 4:**
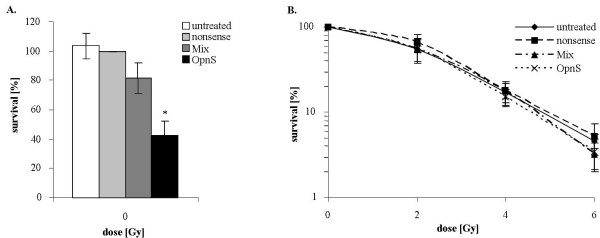
**Clonogenic survival of either non-irradiated or irradiated MDA-MB-231 cells after siRNA transfection**. **A**. Clonogenic survival of MDA-MB-231 cells after transfection. Treatment with just OpnS had a strong effect on clonogenic survival at 0 Gy. The relative clonogenic survival of cells that were transfected with nonsense siRNA was arbitrarily established as 100%. Data represent the average values (± SD) of three independent experiments (* p < 0.05, ** p < 0.001). **B**. Clonogenic survival after transfection with OPN-specific siRNA (Mix, OpnS) in combination with irradiation at 2, 4 or 6 Gy. To examine the additional effects of irradiation all values of clonogenic survival at 0 Gy were set arbitrarily at 100%. Cells transfected with OpnS showed an increased radiosensitivity. After irradiation at 6 Gy, a dose modifying factor (DMF_10_) of 1.1 and an enhancement factor of 1.5 (p = 0.09) were calculated for the siRNA construct OpnS. Data represent the average values (± SD) of three independent experiments.

We demonstrated that incubation with siRNA OpnS is more effective to reduce the clonogenic survival of MDA-MB-231 cells than incubation with siRNA Mix. In particular, we found that transfection with OpnS significantly decreased the clonogenic survival to 42% (p = 0.008) (Fig. 4A.). In contrast, transfection with Mix was ineffective at reducing the clonogenic survival (82%) (p = 0.4).

Irradiation of MDA-MB-231 cells at 2 Gy reduced the clonogenic survival to 60% (SF_2 _= 0.60) (data not shown). The combination of treatment with OpnS siRNA and irradiation also reduced the clonogenic survival as compared to single siRNA treatment. Incubation with OpnS, and additional irradiation at 2 Gy significantly decreased the clonogenic survival to 30% (p < 0.001). Furthermore, with higher irradiation dose transfection with OpnS resulted in a weak radiosensitization with a DMF_10 _of 1.1 and an enhancement factor of 1.5 at 6 Gy (p = 0.09) (Fig. 4B.).

## Discussion

It is well known that intratumoral and plasma levels of the phosphoprotein OPN are increased in many tumors such as lung cancer [21], esophageal cancer [22], prostate cancer [23], glioma [24], soft tissue sarcoma [25] and breast cancer [5,14]. Furthermore, it has been shown that an elevated OPN level is associated with poor prognosis for cancer patients [5,6,12,14,15]. In addition, different studies have found that high OPN levels are associated with poor response to conventional treatment modalities including radiotherapy (reviewed in [9]). However, little is known about the relationship between OPN expression and radiosensitivity.

Our analyses demonstrate that both Mix and OpnS siRNAs (Table 1) are suitable to clearly reduce mRNA levels of OPN (Fig. 1A.). Furthermore, we detected a clear decrease of extracellular OPN protein levels after transfection with OPN siRNA (Fig. 1B.). In contrast, the intracellular OPN protein level was only partially decreased after transfection with OPN siRNA. However, intracellular OPN was detected at a higher molecular weight range (64 kDa) as compared with extracellular OPN that was detected at 50 kDa. The molecular weight difference may represent post-translational modifications such as glycosylation, phosphorylation and sulfatization [4,26,27]. In addition, there is evidence from the literature that two forms of OPN exist: a secreted form (sOPN) and an intracellular form (iOPN). Shinohara and co-workers [28] proposed that sOPN and iOPN represent alternative translational products of a single full-length OPN mRNA that have a molecular weight difference of 5 kDa. In contrast to sOPN, the iOPN protein lacks a signal peptide, which allows the iOPN protein to localize to the cytoplasm but not to the Golgi apparatus [28]. Furthermore, it has been shown that extracellular OPN is important for bone marrow cell activation and the subsequent outgrowth of distant tumors [19], and it also affects the cellular response and increases lung metastasis in mice that have received cells preincubated with OPN [29].

The siRNA transfection showed clear effects on different cellular parameters. Treatment with OpnS resulted in a clear reduction of clonogenic survival, inhibition of migration and increased rate of apoptosis (Fig. 2, 3, 4A.), whereas treatment with the siRNA construct Mix caused an obvious reduction in the rate of migration. However, no differential effects were found with respect to apoptosis and clonogenic survival. The different effects of OpnS and Mix on clonogenic survival and apoptosis frequency are possibly caused by the different sequences that are recognized by the siRNAs. Possibly, OPN RNA sequences are not assessable in the same way by the different siRNAs. Mix is a pool of four siRNAs and might cause more off-target effects than OpnS which could reverse the original effects. We chose the siRNA technology for transient inhibition of OPN expression in MDA-MB-231 cells. A disadvantage of the siRNA technology is that it is not possible to reach a permanent reduction of OPN expression. However, in vitro it is an efficient method to knockdown OPN.

Taken together the effects of OPN inhibition are in agreement with previous findings that the knockdown of OPN reduces the clonogenic survival, migration and invasion rate, and proliferation in different breast cancer cell lines [30-32]. Furthermore, various studies have demonstrated the effects of OPN silencing or OPN overexpression on several downstream elements of OPN in Western blot analysis. In particular, Tuck and co-workers [33,34] found an induction of uPA expression in response to OPN treatment and an association of uPA expression with OPN-induced invasion and migration in human breast cancer cells. These findings are consistent with our data analyzing the protein expression levels of the migration marker uPA with ELISA in cell lysates of MDA-MB-231 cells that showed a clear, albeit not significant, reduction of uPA protein levels after transfection with OPN siRNAs and irradiation (data not shown). Other investigators have demonstrated that knockdown of OPN decreases the expression of PI3'-kinase, JNK1/2, Src and Akt, uPA, MMP-2 and -9 in various tumor cell lines [35-39].

In the present study, for the first time we were able to demonstrate that OPN silencing affects the radiobiological behavior of human cancer cells. Moreover, we found that OPN knockdown by OPN siRNA could very effectively decrease OPN mRNA and protein levels after additional irradiation (Fig. 1). Furthermore, an additional decrease in the intracellular OPN protein level was detected in Western blot analyses after irradiation (Fig. 1C.). However, another study analyzed the effect of radiation on OPN levels in osteoblastic cells and found a slightly elevated expression of OPN on days 14 and 21 after irradiation [40].

Moreover, the additional irradiation at 2 Gy caused a significant reduction in the rate of migration (Fig. 2B.). We demonstrated that treatment with OpnS resulted in a significant increase in irradiation-induced apoptosis (Fig. 3B.). This is in agreement with Lee and co-workers [41], who showed that treatment with recombinant OPN confers an increased resistance to UV-induced apoptosis in HT29 cells [41]. However, OPN siRNA transfection alone and in combination with irradiation showed only minor effects on apoptosis compared with effects on clonogenic survival. Possibly the MAA (methoxyacetic acid) assay can reflect a better correlation because besides apoptosis this assay determines other modes of cell death such as micronucleation or multinucleated cells [42].

To our knowledge, this is the first study demonstrating that knockdown of OPN influences the radiosensitivity of cancer cells. OPN knockdown even caused a weak radiosensitization with a higher irradiation dose (Fig. 4B.). Considering the non-significant effects on radiosensitivity *in vitro *it appears that OPN siRNA treatment predominantly affects clonogenicity and migration rate. However, *in vivo *we cannot be sure that siRNAs would find their target molecules and concentrate as it would be appropriate in solid tumors. Therefore, a combined treatment of siRNAs with irradiation might be necessary. Another study which analyzed the influence of OPN silencing confirmed the impact of OPN expression on the efficacy of irradiation. Solberg and co-workers [43] found that irradiation of xenograft tumors in mice induces the expression of mouse VEGF (mVEGF) and mouse OPN (mOPN), which are both closely associated with angiogenesis. Moreover, the expression of mOPN was directly proportional to the mVEGF levels in tumors which indicates that mOPN can serve as an alternative marker of tumor recovery after radiotherapy. Furthermore, clinical studies have found that elevated OPN levels are associated with poor prognosis in head and neck cancer [9,12,13,44-47] and breast cancer [3,48].

## Conclusions

In summary, in the present study we were able to demonstrate for the first time that an OPN knockdown combined with irradiation has additive effects on clonogenic survival, migration and the induction of apoptosis. Furthermore, we showed that silencing of OPN with siRNA causes a weak radiosensitization of MDA-MB-231 cells. This suggests that OPN is an attractive target to improve the efficacy of radiotherapy. Additional radiobiological studies are necessary to investigate the role of OPN and its association with radiosensitivity of other tumor cell lines.

## Competing interests

The authors declare that they have no competing interests.

## Authors' contributions

AH designed the study, performed experimental procedures, analyzed the data and drafted the manuscript. HW, MKa, HT and DV aided in study design, analyzed the data and reviewed the manuscript. MKo performed experimental procedures, analyzed the data and reviewed the manuscript. MB designed the study, analyzed the data and drafted the manuscript. All authors read and approved the final manuscript.
